# Activation of NLR family, domain of pyrin containing 3 inflammasome by nitrous oxide through thioredoxin-interacting protein to induce nerve cell injury

**DOI:** 10.1080/21655979.2021.1954741

**Published:** 2021-08-04

**Authors:** WenJuan Liu, GuangMing Zhang, Bo Sun, ShuYan Wang, YinZhong Lu, Hong Xie

**Affiliations:** a Department of Anesthesiology, The Second Affiliated Hospital of Soochow University, Suzhou City, Jiangsu Province, China; b Department of Anesthesiology, Tongren Hospital, Shanghai Jiaotong University School of Medicine, Shanghai City, China

**Keywords:** N_2_O, txnip, nlrp3, nerve cell injury

## Abstract

Nitrous Oxide (N_2_O) has been shown to be neurotoxic, but its specific mechanism is still unclear. The purpose of this work is to probe into the impact of N_2_O on nerve cell injury through regulating thioredoxin-interacting protein (TXNIP)/the NOD-like receptor domain of pyrin containing 3 (NLRP3) pathway. The results indicated that, N_2_O exposure elevated TXNIP/NLRP3 expression *in vivo* and *in vitro*, led to declined learning and memory capabilities in mice, reduced apoptosis rate in hippocampal neuron and Nissl bodies, elevated inflammatory factors TNF-α, IL-1β and IL-6 levels, as well as cleaved caspase-3 and Bax expressions, and reduced Bcl-2 expression. Overexpressing TXNIP or NLRP3 further aggravated these injuries, but knocking down TXNIP or NLRP3 improved them. CO-IP indicated that TXNIP and NLRP3 can be combined, with interaction relationship. All in all, the results manifested that N_2_O is available to promote nerve cell inflammation and apoptosis through activating the TXNIP/NLRP3 pathway that can be used as a potential target for N_2_O-induced nerve damage in the future.

## GRAPHICAL ABSTRACT

**Figure:**
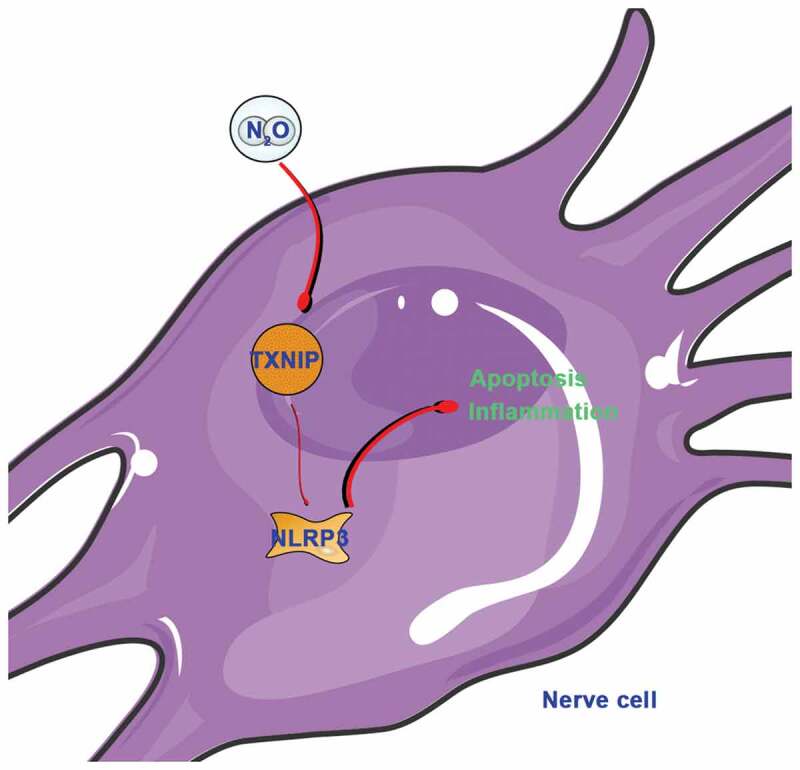

## 1. Introduction

Nitrous oxide (N_2_O), a colorless and odorless gas, is commonly applied for human medicine as anesthetics [1]. But due to its low anesthesia efficiency, N_2_O cannot provide deep anesthesia during surgery. Therefore, it is often clinically used combined with other anesthetics [2]. In recent years, many studies have reported that N_2_O as an inhalational anesthesia gas damages nerve cell. Reports from Taiwan indicate that patients exposed to N_2_O may result in severe damage to the nerves of lower limbs, thus affecting sports [3]. Based on recent studies, N_2_O damages rat nervous system and inhibits neuronal axon regeneration [4]. Additionally, JT V et al. reported that long-term intake of N_2_O may induce neuronal apoptosis in rat brain tissue [5]. Although it has been revealed that N_2_O damages the nervous system, its specific mechanism has not been fully elucidated.

Thioredoxin-interacting protein (TXNIP) gene located on human chromosome 1q21.1, belongs to α-arrestin protein family [6]. In reference to a great many studies, TXNIP participates in regulating oxidative stress, inflammation, vascular dysfunction and cellular stress in neurological diseases [7–9]. Highly expressed TXNIP is found in neurons, microglia and astrocytes, which can promote Bax and cleaved caspase-3 expressions in cells to induce apoptosis [10–12]. Additionally, high-level TXNIP has also been found in acute neurological diseases and neurodegenerative diseases. In artery occlusion animal models, the researchers found that the cerebral infarction area with TXNIP knockout decreased by about 40% [13]. In Parkinson’s disease, researchers have found that overexpressing TXNIP is available to elevate α-synuclein and block autophagy, thereby exacerbating Parkinson’s disease development [14]. It is often necessary to consider the interaction of TXNIP and NLR family, domain of pyrin containing 3 (NLRP3) in neurological diseases. Excessive TXNIP will combine with TRX to aggravate oxidative stress, and with NF-κB to activate the inflammatory complex associated with NLRP3, thus further strengthening inflammation and promote the activation of caspase-1 to induce apoptosis [15–18]. In addition, NLRP3 and caspase-1-induced regulatory necrosis are classified as pyroptosis. This type of apoptosis is accompanied by damage to the plasma membrane of the cell, leading to cell necrosis and dissolution and promoting the rapid release of inflammatory factors from the apoptotic cells, thus triggering local inflammation [19]. Although TXNIP/NLRP3 pathway is a key target in nerve injury, it is still unclear whether it takes part in the process of N_2_O doing damage to nerve cells.

In our work, via nitrous oxide exposure to establish *in vivo* and *in vitro* nerve injury models, and through knocking down or overexpressing TXNIP/NLRP3 level, it deeply probed into the impact of TXNIP/NLRP3 on N_2_O-induced nerve cell injury from the perspective of apoptosis and inflammation.

## 2. Methods

### 2.1 Animal model establishment

In total 36 7-day-old male C57/BL6 mice purchased from Hunan SJA Laboratory Animal CO., LTD, were kept at 24 ± 2°C and with humidity of 50%-60%, eating and drinking freely. After one-week adaptive feeding, the mice were by random allocated into Control group and N_2_O group (n = 12), later exposed to air (70% nitrogen, 5% CO_2_, and 25% oxygen) or N_2_O (70% N_2_O, 5% CO_2_, and 25% oxygen) for 2 hours a day for 3 days. Gas analyzers (GE Datex 5 Ohmeda; Soma Technology, U.S.) were applied to continuously monitor nitrogen, N_2_O and oxygen concentration in room. Additionally, in order to evaluate the role of TXNIP in N_2_O-induced hippocampal nerve injury, the TXNIP inhibitor TXNIP-IN-1 (10 mg/kg, ChemeGen china) was injected intraperitoneally 24 h before N_2_O exposure (TXNIP-IN-1 group, n = 12). After that, 6 mice in each group were euthanized and part of the brain tissue was collected and fixed in 4% paraformaldehyde, while the rest of the brain tissue was stored at −80°C for further study. After 30 days, other mice in each group received tests for memory and learning capabilities. All animal experiments were conducted in accordance with the requirements of the Animal Care and Use Committee of the Second Affiliated Hospital of Soochow University, and in compliance with the guidelines of the National Institute of Public Health. A timeline of experimental design was shown in Table 1.

**Table 1.** Timeline of experimental design

Time (days)	Control group (n = 12)	N_2_O group (n = 12)	TXNIP-IN-1 group (n = 12)
1			injection of TXNIP-IN-1
2	Exposure to the air for 2 hours a day	Exposure to N_2_O for 2 hours a day	Exposure to N_2_O for 2 hours a day
3			
4			
5	euthanasia of 6 mice and collection of samples	euthanasia of 6 mice and collection of samples	euthanasia of 6 mice and collection of samples
31-35	The remaining 6 mice for MWM experiment	The remaining 6 mice for MWM experiment	The remaining 6 mice for MWM experiment

N_2_O, Nitrous Oxide; TXNIP-IN-1, thioredoxin-interacting protein inhibitor; MWM, Morris Water Maze.

### 2.2 Morris Water Maze (MWM) experiment

The remaining 6 mice in each group were tested in MWM experiment based on the previous study. The test was carried out by an operator who did not know the grouping. The device consists of a circular steel pool (50 cm high, 110 cm in diameter), which was filled with water to the point that was 1 cm higher than the platform (35 cm deep, 12 cm in diameter). The swimming pool covered by blue curtains was located in an isolated room (humidity of 60%, 24°C). The water was made opaque through adding titanium dioxide. For memory test, all mice were given 4 times a day with an interval of 30-40 minutes. Before that, the mice were placed in different starting positions in the water facing the wall. If it cannot find the platform within 1 minute, it was allowed to stay on the platform for 15 s. Via video tracking system to record the swimming activity of each animal and the escape latency, that is, the time from putting it in the water to finding the platform, later the platform was removed from the pool to perform a spatial probe test. The mouse was allowed to swim freely for 2 min before being placed in the opposite quadrant. After recording platform crossing numbers. Our team analyzed the data via motion detection software designed for the MWM experiment (Shanghai Mobile Datum Information Technology Co., Ltd, Shanghai, China).

### 2.3 Terminal deoxynucleotidyl transferase-mediated dUTP-biotin nick end labeling assay (TUNEL)

The mouse hippocampus tissue was taken out of paraformaldehyde to embed in paraffin, and cut into slices (4 μm), which were dewaxed with xylene and rehydrated with graded alcohol. In terms of manufacturer’s protocol, TUNEL staining kits (Keygen Biotech, China) were applied to assess neuronal apoptosis in hippocampal tissue. Under fluorescence microscopes (Olympus, Japan), the total number of TUNEL-positive neurons (apoptotic neurons) in hippocampus was counted.

### 2.4 Nissl staining

The hippocampal slices were stained with 1% toluidine blue (Sigma, China) for 40 minutes to stain Nissl bodies in neurons. Later they were washed with distilled water for 5 minutes, and quickly dehydrated in gradient alcohol. After adding xylene and sealing the slices with neutral glue, they were inspected under microscopes (Nikon Eclipse E100; Nikon Inc., Japan). The number of stained cells was counted at 400 × magnification.

### 2.5 Cell culture

From the brain of the 7-day old normal mice, the hippocampus was separated and cut into small pieces, later digested with 0.125% trypsin at 37°C for 15 minutes. Mouse primary hippocampal neurons were seeded into 10 mm dishes coated with polylysine (10 mmol/L), later ground and centrifuged, at a concentration of 1 × 10^6^ cells/mL in 0.25% Glumax (Gibco), and 2% B27 (Gibco) neural basal medium (Gibco) for cultivation. In order to inhibit glial cell proliferatio, 2.5 μg/mL cytosine arabinoside (Sigma Aldrich, U.S.) was added to the medium, which was changed every three days. For induction of hippocampal cellular damage, after 14-day continuous culture, the cells were exposed to N_2_O (70% N_2_O, 5% CO_2_, and 25% oxygen) for 6 h, via gas analyzers to continuously monitor the gas concentration.

### 2.6 Immunofluorescence staining

After adjusting the hippocampal cells to 1.5 × 10^4^/100 µL, they were centrifuged for 10 minutes to make them adhere to the slides, and later fixed with 4% paraformaldehyde for 15 minutes. The slides were incubated with 0.25% bovine serum albumin for 5 minutes, and incubate with 0.05% Triton X-100 on the slides for 5 minutes. After incubating with anti-VEGF (1: 1,000, ab46154, Abcam, U.S.), anti-βIII tubulin (1: 1000, G7121, Promega) at 4°C overnight, the slides mixed with green fluorescence-labeled goat anti-rabbit secondary antibody (1: 1000, sc-2012, Santa Cruz, U.S.) were placed in the darkness at 37°C for 60 minutes, where the nucleus was stained with 4ʹ,6-Diamidino-2-phenylindole (DAPI) for 10 minutes, later the slides observed under fluorescence microscopes (Leica, Germany).

### 2.7 Cell transfection

Small interfering RNA (siRNA) targeting TXNIP and NLRP3, TXNIP overexpression plasmid and its NC were designed and synthesized by GenePharma (China). In order to up-regulate NLRP3, NLRP3 adenovirus vector was purchased from Genomeditech (China). The above reagents were transfected into hippocampal neurons via Lipofectamine 2000 (Thermo Scientifc, U.S.) in reference to the instructions.

### 2.8 Flow Cytometry

Annexin V-FITC (AV-FITC) Apoptosis Detection Kit (Dojindo) was applied to detect the apoptosis in hippocampal neurons, which were collected to adjust to a concentration of 1 × 10^6^ cells/mL with cold D-Hanks buffer. After adding propidium iodide (PI, 10 μL) and AV-FITC (10 μL) to cell suspension (100 μL), they were incubated in the darkness at room temperature for 15 minutes. It was analyzed to check cell apoptosis rate via flow cytometry.

### 2.9 Enzyme-linked immunosorbent assay (ELISA)

In terms of the instructions, ELISA kits were applied to detect TNF-α, IL-1β and IL-6 levels in brain tissues and cells, all the kits purchased from Shanghai Enzyme-linked Biotechnology Co., Ltd.

### 2.10 Reverse transcription quantitative polymerase chain reaction (RT-qPCR)

Based on the instructions, RNA easy Mini kits (Qiagen, U.S.) was applied to extract total RNA from the cultured cells. After RNA quantification, reverse transcription was performed via high-capacity reverse transcription kits (Applied Biosystems, U.S.). Real-time PCR was performed and repeated three times on ABI Prism 7900HT instruments (Applied Biosystems) via Power SYBR Green PCR Master Mix (Life Technologies, U.S.). mRNA expression was normalized in reference to GAPDH gene expression. The gene expression level was calculated in accordance with 2^−ΔΔCt^ (the primer sequence in Table 2).

**Table 2.** RT-qPCR primer sequence

	Primer sequences (5′ - 3′)
GAPDH	Forward: 5ʹ-CCTCGTCTCATAGACAAGATGGT-3’
	Reverse: 5ʹ- ACCTCAGTGTAAGTGGGTGG-3’
TXNIP	Forward: 5ʹ-GATACCCCAGAAGCTCCTCC-3’
	Reverse: 5ʹ-AACGCTTCACGAATTTGCGT-3’
NLRP3	Forward: 5ʹ- AGCCTTCCAGGATCCTCTTC-3’
	Reverse: 5ʹ- CTTGGGCAGCAGTTTCTTTC-3’

### 2.11 Western blot

Via RIPA lysis buffer (Beyotime) to extract total protein samples from hippocampal tissue or cells, later the protein samples were loaded and separated with 10% SDS-PAGE to transfer to PVDF films. After blocking with 5% skim milk for 1 hour, the films were incubated with the following primary antibodies: Bax (ab32503, Abcam), Bcl-2 (12,789-1-AP, Proteintech), cleaved caspase-3 (ab2302, Abcam), TXNIP (ab188865, Abcam), NLRP3 (ab214185, Abcam), and GAPDH (60,004-1-Ig, Proteintech), later incubated with horseradish peroxidase-conjugated secondary antibody (Abcam). Enhanced chemiluminescence kits (Pierce, U.S.) were used to generate the blot, GAPDH as the internal reference.

### 2.12 Co-immunoprecipitation (Co-IP) analysis

Co-IP analysis was performed via Capture and Release Reversible Immunoprecipitation System Kits (Merck Millipore, U.S.). The antibodies applied for Co-IP determination were in the following: TXNIP antibody (ab188865, Abcam) and NLRP3 (ab214185, Abcam), rabbit/mouse IgG as the negative control for Co-IP reaction.

### 2.13 Data analysis

The experimental results were expressed as mean ± standard deviation (SD), via SPSS 22 software for data analysis, including Student’s T test and one-way analysis of variance (ANOVA). Via Tukey’s test to perform multiple variance correction on the samples, the difference between the experimental groups was considered significant when *P* < 0.05.

## 3. Results

### 3.1 N_2_O-induced nerve cell injury in vivo was relative to regulating TXNIP/NLRP3

**Figure 1.**
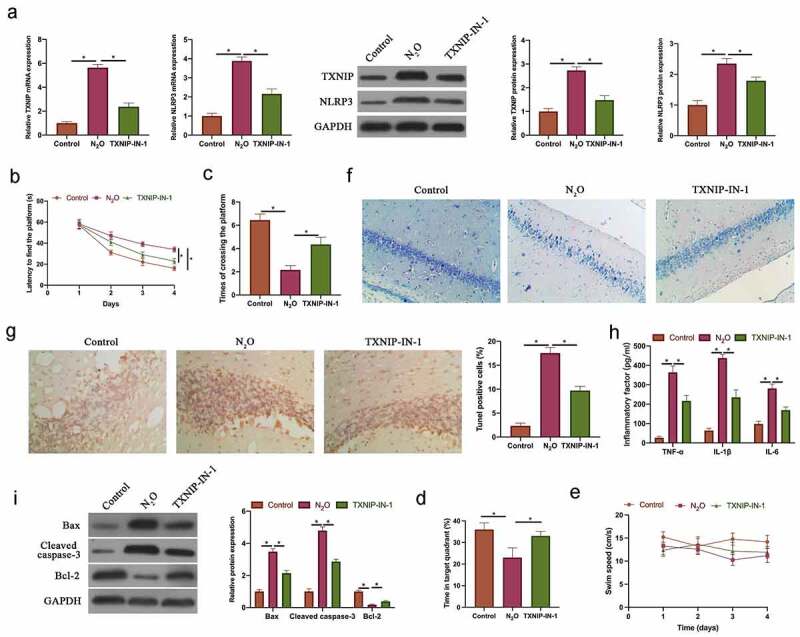
N_2_O-induced nerve cell injury in vivo was relative to regulating TXNIP/NLRP3

To probe into the role of TXNIP in N_2_O-induced neurocyte injury, TXNIP in mice was knocked down through injecting TXNIP-IN-1 before N_2_O exposure. N_2_O exposure signally increased TXNIP and NLRP3 expressions, while knocking down TXNIP visually reduced them (Figure 1a, *p < 0.05*). MWM experiment revealed that N_2_O exposure elevated escape latency and reduced the number of platform crossings, whereas knocking down TXNIP reversed this phenomenon (Figure 1b & Figure 1c, *p < 0.05*). The time spent in the target quadrant in mice was reduced through N_2_O exposure, but increased via knockdown of TXNIP (Figure 1d, *p < 0.05*). The pathological damage in neurons was detected via Nissl staining, which revealed that N_2_O exposure reduced the number of Nissl bodies, while knocking down TXNIP restored it (figure 1f). Additionally, apoptosis in hippocampal neurons was examined via TUNEL staining and western blot. N_2_O exposure obviously elevated hippocampal neuron apoptosis rate, promoted Bax and Cleaved caspase-3 expressions, and decreased Bcl-2 expression (Figure 1g & Figure 1i *P < 0.05*). But after knocking down TXNIP, hippocampal neuron apoptosis was signally suppressed. Subsequently, pro-inflammatory cytokine levels were further checked by ELISA. N_2_O exposure increased TNF-α, IL-6, and IL-1β levels in the brain group; after knocking down TXNIP, inflammation in brain tissue was observably suppressed (Figure 1h, *p < 0.05*). These findings indicated that N_2_O was available to induce neurocyte injury through regulating TXNIP/NLRP3.

A: RT-qPCR and western blot to check TXNIP/NLRP3 mRNA and protein expressions in hippocampus in Control, N2O, and TXNIP-IN-1 groups; B: MWM to detect escape latency in Control, N2O, and TXNIP-IN-1 groups; C: MWM to test platform crossing times in Control, N2O, and TXNIP-IN-1 groups; D: MWM to test the time spent through the target quadrant in mouse; E: MWM to record the swimming speed of the mice; F: Nissl staining to check number of Nissl bodies in hippocampal tissue in Control, N2O, and TXNIP-IN-1 groups; G; TUNEL staining to check hippocampus neuron apoptosis rate in Control, N2O, and TXNIP-IN-1 groups; I: Western blot to detect Bax, cleaved caspase-3 and Bcl-2 expressions in Control, N2O, and TXNIP-IN-1 groups; I: ELISA to detect TNF-α, IL-1β and IL-6 levels in hippocampus in Control, N2O, and TXNIP-IN-1 groups. Values were expressed as mean ± SD (n = 6) display; one-way ANOVA was applied to calculate the significance of each group; the variance was corrected using Tukey’s test. *P < 0.05.

### 3.2 N_2_O induced neuronal injury in vitro

**Figure 2.**
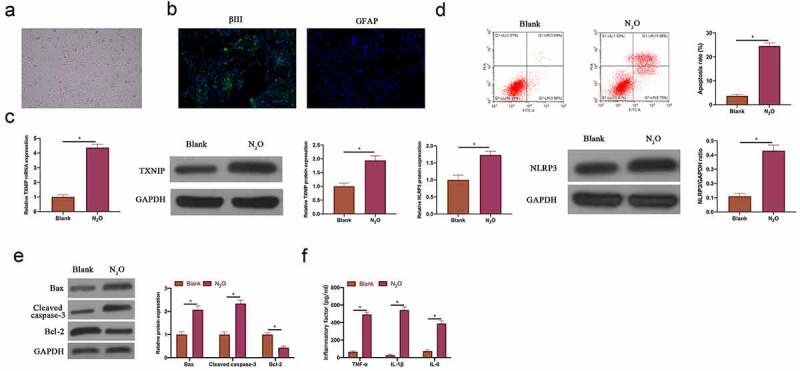
N_2_O induced neuronal injury *in vitro.*

Next, the impact of N_2_O on neuronal injury was further explored through *in vitro* experiments. Firstly, primary hippocampal neurons were isolated from mice. The isolated primary hippocampal neurons had long synapses (Figure 2a). Additionally, immunofluorescence revealed (Figure 2b) that the neuron-specific protein βIII tubulin was positively expressed in the isolated cells, whereas the glial cell-specific protein GFAP was negatively expressed, indicating that the isolated cells were hippocampal neurons instead of glial cells. After N_2_O exposure, TXNIP expression and NLRP3 in hippocampal neurons elevated signally (Figure 2c, *p < 0.05*). The findings in flow cytometry and western blot revealed that N_2_O exposure promoted hippocampal neuron apoptosis rate, increased Bax and cleaved caspase-3 expressions and decreased Bcl-2 expression (Figure 2d & Figure 2e, *P < 0.05*). In addition, N_2_O visually elevated TNF-α, IL-1β and IL-6 levels in hippocampal neurons (figure 2f, *p < 0.05*). This indicates that N_2_O is available to induce damage to hippocampal neurons.

A: Representative images of hippocampal neurons; B: Immunofluorescence to detect βIII tubulin and GFAP protein expression in hippocampal neuron cells; C: RT-qPCR to detect TXNIP/NLRP3 mRNA and protein expression in hippocampal neurons in Blank and N2O groups; D: Flow cytometry to detect hippocampal neuron apoptosis in Blank and N2O groups; E: Western blot to detect Bax, cleaved caspase-3 and Bcl-2 protein expressions in hippocampal neurons in Blank and N2O groups; F: ELISA to test TNF-α, IL-1β and IL-6 levels in hippocampal neurons in Blank and N2O groups. Values were expressed as mean ± SD (n = 3) display; one-way ANOVA was applied to calculate the significance of each group; the variance was corrected using Tukey’s test. *P < 0.05.

### 3.3 Overexpressing TXNIP or NLRP3 promoted N_2_O-induced hippocampal neuron injury

**Figure 3.**
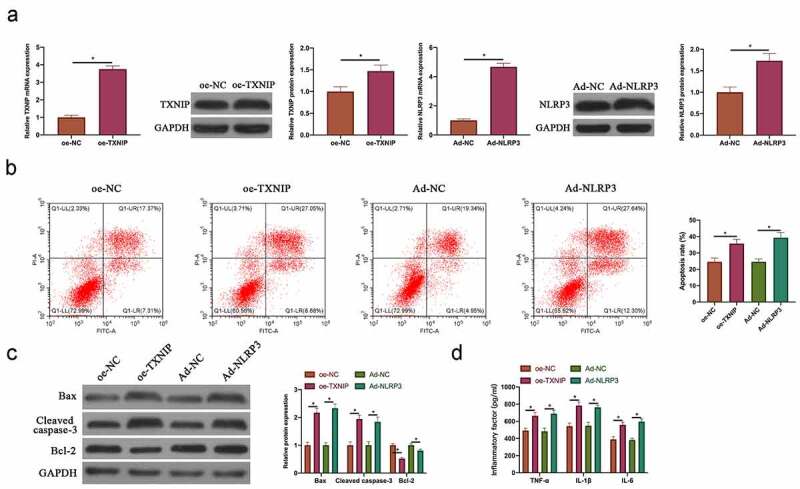
Overexpressing TXNIP or NLRP3 promoted N_2_O-induced hippocampal neuron injury

Next, TXNIP overexpression plasmid and NLRP3 adenovirus vector were transfected into N_2_O-exposed hippocampal neurons. After transfection, TXNIP and NLRP3 expressions elevated signally (Figure 3a, *p < 0.05*). Additionally, the findings in flow cytometry and western blot revealed that overexpressing TXNIP and NLRP3 both increased hippocampal neuron apoptosis rate and Bax and cleaved caspase-3 expression, whereas decreased Bcl-2 expression (Figure 3b & Figure 3c, *P < 0.05*). Overexpressing TXNIP and NLRP3 also elevated TNF-α, IL-1β and IL-6 levels (Figure 3d, *p < 0.05*). Taken together, these findings indicated that overexpressing TXNIP and NLRP3 was available to promote N_2_O-exposed hippocampal neuron injury.

A: RT-qPCR and western blot to detect the impact of overexpressing TXNIP and NLRP3 on TXNIP/NLRP3 mRNA and protein expression in hippocampal neurons; B: Flow cytometry to detect the impact of overexpressing TXNIP and NLRP3 on hippocampal neuron apoptosis; C: Western blot to detect the impact of overexpressing TXNIP and NLRP3 on Bax, cleaved caspase-3 and Bcl-2 protein expressions in hippocampal neurons; D: ELISA to detect the impact of overexpressing TXNIP and NLRP3 on TNF-α, IL-1β and IL-6 levels in hippocampal neurons. Values were expressed as mean ± SD (n = 3) display; one-way ANOVA was applied to calculate the significance of each group; the variance was corrected using Tukey’s test. *P < 0.05.

### 3.4 Silencing TXNIP or NLRP3 inhibited N_2_O-induced hippocampal neuron injury

**Figure 4.**
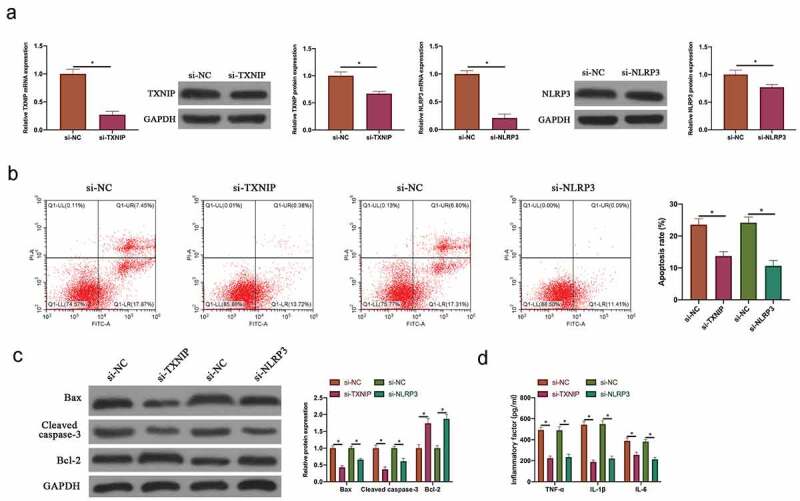
Silencing TXNIP or NLRP3 inhibited N_2_O-induced hippocampal neuron injury

To silence TXNIP and NLRP3 expressions in N_2_O-exposed hippocampal neurons, siRNA targeting TXNIP and NLRP3 were transfected into neurons. After transfecting siRNA, TXNIP and NLRP3 expressions in hippocampal neurons were visually reduced (Figure 4a, *p < 0.05*). Additionally, after silencing TXNIP and NLRP3, N_2_O-exposed hippocampal neuron apoptosis rate and Bax and cleaved caspase-3 expressions were signally reduced, whereas Bcl-2 expression was visually elevated (Figure 4b & Figure 4c, *P < 0.05*). Based on ELISA findings, after silencing TXNIP and NLRP3, TNF-α, IL-1β and IL-6 levels in N_2_O-exposed hippocampal neurons were obviously reduced (Figure 4d, *p < 0.05*). Taken together, these findings indicated that silencing TXNIP and NLRP3 was available to protect N_2_O-induced hippocampal neuron injury.

A: RT-qPCR and western blot to detect the impact of silencing TXNIP and NLRP3 on TXNIP/NLRP3 mRNA and protein expression in hippocampal neurons; B: Flow cytometry to detect the impact of silencing TXNIP and NLRP3 on hippocampal neuron apoptosis; C: Western blot to detect the impact of silencing TXNIP and NLRP3 on Bax, cleaved caspase-3 and Bcl-2 protein expressions in hippocampal neurons; D: ELISA to detect the impact of silencing TXNIP and NLRP3 on TNF-α, IL-1β and IL-6 levels in hippocampal neurons. Values were expressed as mean ± SD (n = 3) display; one-way ANOVA was applied to calculate the significance of each group; the variance was corrected using Tukey’s test. *P < 0.05.

### 3.5 TXNIP/NLRP3 axis participated in N_2_O-induced hippocampal neuron injury

**Figure 5.**
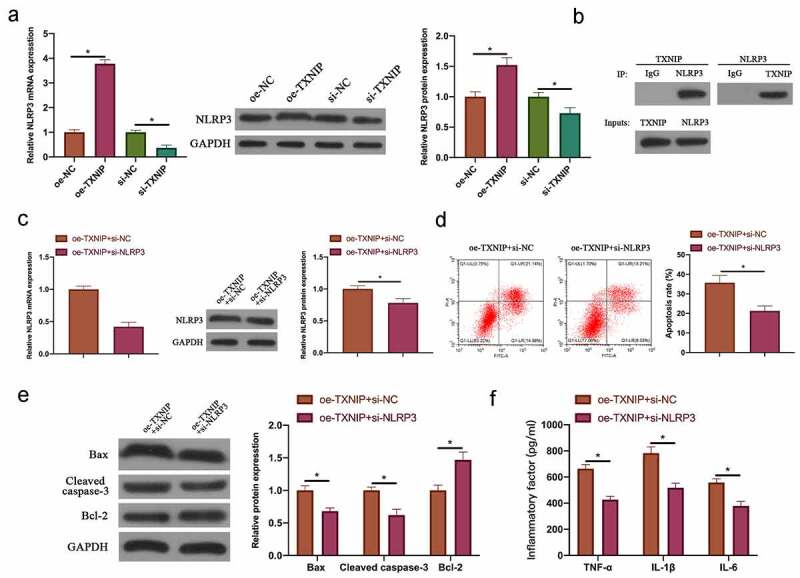
TXNIP/NLRP3 axis participated in N_2_O-induced hippocampal neuron injury

Next, our team examined the relationship between N_2_O-induced hippocampal neuron injury and TXNIP/NLRP3. The findings revealed that after overexpressing TXNIP or silencing, NLRP3 expression in hippocampal neurons was also up-regulated or down-regulated (Figure 5a, *p < 0.05*). In reference to Co-IP findings, TXNIP reduced NLRP3 protein expression, and NLRP3 also reduced TXNIP expression (Figure 5b), which indicated that TXNIP was available to activate NLRP3 inflammasomes in hippocampal neuronal injury. Next, through co-transfecting oe-TXNIP and si-NLRP3 to probe into the mechanism of TXNIP/NLRP3 in regulating hippocampal neuron injury, the promotion of transfecting oe-TXNIP on NLRP3 expression was reversed by transfecting si-NLRP3 (Figure 5c, *p* < 0.05). In reference to the findings in flow cytometry and western blot (Figure 5d & Figure 5e, *P < 0.05*), the impact of overexpressing TXNIP on hippocampal neuron apoptosis as well as Bax and cleaved caspase-3 protein expressions was signally reversed through transfecting si-NLRP3. Additionally, co-transfecting si-NLRP3 also reversed the promotion of overexpressing TXNIP on TNF-α, IL-1β and IL-6 levels in hippocampal neurons (figure 5f, *p < 0.05*).

A: RT-qPCR and western blot to detect NLRP3 mRNA and protein expression in hippocampal neurons after overexpressing or silencing TXNIP; B: Co-IP to detect the interaction between TXNIP and NLRP3; C: RT-qPCR and western blot to detect NLRP3 mRNA and protein expression in hippocampal neurons after co-transfecting oe-TXNIP and si-NLRP3; D: Flow cytometry to detect hippocampal neuron apoptosis rate after co-transfecting oe-TXNIP and si-NLRP3; E: Western blot to detect Bax, Blc-2 and cleaved caspase-3 protein expressions in hippocampal neurons after co-transfecting oe-TXNIP and si-NLRP3; F: ELISA to detect TNF-α, IL-1β and IL- 6 levels in hippocampal neurons after co-transfecting oe-TXNIP and si-NLRP3. Values were expressed as mean ± SD (n = 3) display; one-way ANOVA was applied to calculate the significance of each group; the variance was corrected using Tukey’s test. *P < 0.05.

## 4. Discussion

N_2_O abuse is neurotoxic and can result in symptoms like acute vitamin B (12) deficiency, pulmonary embolism, acute psychosis and peripheral neuropathy [20–22]. But the mechanism by which it affects neurological diseases is still not clear. In this work, we found that N_2_O is available to activate NLRP3 inflammasome through increasing TXNIP expression to promote hippocampal neuronal apoptosis and inflammatory damage.

In recent years, increasing studies have supported that abusing anesthetics may result in damage to the nervous system, which mainly depends on the dose, type and volume of the anesthetics [23]. In our work, 70% of N_2_O exposure is available to result in neuronal apoptosis, which is consistent with the findings of previous studies [24]. But N_2_O at lower concentration cannot provide sufficient paralysis for the nervous system, so combining N_2_O with different anesthetics may be a feasible way. It is worth noting that most of the patients who are frequently exposed to N_2_O are recreational users. N2O as a leisure and recreational drug is used more and more frequently. Studies have revealed that N2O lifetime prevalence in the United Kingdom and the United States is 38.6% and 29.4% [25], respectively. Additionaly, due to the lack of medical advice, N2O abuse is available to lead to more serious consequences, like death or cardiovascular complications [26,27]. Xenon has been applied to improve neurotoxicity resulting from N2O [28]. Based on transcriptomics data, xenon pretreatment reverses 49 N2O-upregulated genes in brain tissue. These genes help explain the molecular mechanism of N2O affecting the brain nervous system [29]. This work found that N2O is also available to affect TXNIP/NLRP3 expression in hippocampus, thereby affecting neuronal apoptosis and inflammation. This provides more evidence to explain N2O neurotoxicity. But the functions of more genes need further exploration. It is noticed that a new type of TXNIP inhibitor was used in this study. TXNIP in the brain tissue was decreased by ventricular lentiviral vector injection or plasmid vector injection in plenty of studies. However, brain surgery can cause damage to animals, leading to their death. A previous study has shown that siTXNIP injection in the ventricle can make the amount of TXNIP protein reduced by nearly six times the size of it [12]. Our study showed that intraperitoneal injection of TXNIO-IN-1 made TXNIP decreased 1.84 times. Although intraperitoneal injection of TXNIP-IN-1 is not as effective as intracerebral injection of siTXNIP, it can greatly reduce animal mortality. This may be due to the fact that the blood brain barrier causes the content of TXNIP-IN-1 entering the ventricle not high, and it is necessary to check the ability of TXNIP-IN-1 entering the blood-brain barrier in the future job.

N2O exposure can cause nerve damage, resulting in impaired spatial learning and memory [30]. In this study, through MWM experiment, we found that N2O exposure increased the escape latency and reduced the number of platform crossing times, which was consistent with previous studies. This has an important relationship with neuronal apoptosis induced by N2O exposure. Neurons are important cells in the cerebral cortex that controls the body’s behavior and memory. A previous study showed that N2O exposure resulted in inhibition of cAMP/cyclic AMP response element-binding protein (CREB) signals in rat hippocampal tissues [31]. The expression of cAMP/CREB is related to neuronal apoptosis, and the up-regulation of cAMP and phosphorylated CREB can promote neuronal Bcl-2 expression, thus decreasing neuronal apoptosis. Recently, Sbai O et al. found that TXNIP affects phosphorylation of CREB in Schwann cells [32], which suggests that TXNIP may accelerate hippocampal neuronal apoptosis through influence of the cAMP/CREB pathway. In this study, hippocampal neuronal apoptosis and inflammation were knocked down by decline of TXNIP. We hypothesized that N2O led to NF-κB nuclear ectopic performance by binding TXNIP to NLRP3 inflammasome, causing the massive release of inflammatory factors and affecting the activation of cAMP/CREB pathway, thereby inducing hippocampal neuronal injury, which needs to be further explored in the future. In addition, some studies have shown that N2O can affect axon dysfunction of motor neurons [33,34]. N2O exposure results in impaired axon regeneration in damaged neurons. Therefore, the abuse of N2O may cause long-term and irreversible damage to neurons and movement. Lately, two studies have shown that the depression of the activation of NLRP3 inflammasomes can be beneficial for axonal regeneration of nerve cells [35,36]. This suggests that the therapeutic effect of TXNIP/NLRP3 knockdown on N2O-induced nerve injury may be connected with axonal regeneration, which needs to be further explored in the future. In reference to increasing studies, anesthetics affect diseases through regulating the TXNIP/NLRP3 signaling pathway. Ma M et al. found that sevoflurane pretreatment is available to inhibit oxygen-glucose deprivation-induced cardiomyocyte injury through regulating TXNIP [37]. Additionally, studies have reported that dexmedetomidine prevents renal ischemia and reperfusion injury through inhibiting the activation of p-38-MAPK/TXNIP [38]. In our work, it was also found that anesthetic N_2_O affects the activation of TXNIP/NLRP3, but N_2_O is more toxic, which promotes TXNIP/NLRP3 expression in hippocampal neurons.

## 5. Conclusion

In summary, this work explored the molecular mechanism of N_2_O toxicity to hippocampal neurons *in vivo* and *in vitro*, which activates NLRP3 expression through TXNIP, thus increasing inflammation level and promoting cell apoptosis. Our findings imply the need to further manage N_2_O abuse to prevent its lifelong injury. Additionally, it provides more data support for TXNIP/NLRP3 as a potential target for treating neurological diseases.
